# The Relative Contribution of Immigration or Local Increase for Persistence of Urban Schistosomiasis in Salvador, Bahia, Brazil

**DOI:** 10.1371/journal.pntd.0003521

**Published:** 2015-03-16

**Authors:** Ronald E. Blanton, Lúcio M. Barbosa, Eliana A. Reis, Theomira M. Carmo, Cláudio R. A. dos Santos, Jackson M. Costa, Peace T. Aminu, Walter A. Blank, Renato Barbosa Reis, Isabel C. Guimarães, Luciano K. Silva, Mitermayer G. Reis

**Affiliations:** 1 Case Western Reserve University, Centre for Global Health and Diseases, Cleveland, Ohio, United States of America; 2 Gonçalo Moniz Research Center, Oswaldo Cruz Foundation, Salvador, Bahia, Brazil; 3 Bahiana School of Medicine and Public Health, Salvador, Bahia, Brazil; 4 Post-graduate Program in Regional and Urban Development, UNIFACS (Universidade Salvador), Imbuí, Salvador, Bahia, Brazil; 5 Center for Control of Zoonoses, Municipal Secretariat of Health, Salvador, Bahia, Brazil; 6 Federal University of Bahia Faculty of Medicine, Sede Mater Praça XV de novembro, s/n—Largo do Terreiro de Jesus, Salvador, Bahia, Brazil; George Washington University Medical Center, UNITED STATES

## Abstract

Urbanization is increasing across the globe, and diseases once considered rural can now be found in urban areas due to the migration of populations from rural endemic areas, local transmission within the city, or a combination of factors. We investigated the epidemiologic characteristics of urban immigrants and natives living in a neighborhood of Salvador, Brazil where there is a focus of transmission of *Schistosoma mansoni*. In a cross-sectional study, all inhabitants from 3 sections of the community were interviewed and examined. In order to determine the degree of parasite differentiation between immigrants and the native born, *S*. *mansoni* eggs from stools were genotyped for 15 microsatellite markers. The area received migrants from all over the state, but most infected children had never been outside of the city, and infected snails were present at water contact sites. Other epidemiologic features suggested immigration contributed little to the presence of infection. The intensity and prevalence of infection were the same for immigrants and natives when adjusted for age, and length of immigrant residence in the community was positively associated with prevalence of infection. The population structure of the parasites also supported that the contribution from immigration was small, since the host-to-host differentiation was no greater in the urban parasite population than a rural population with little distant immigration, and there had been little differentiation in the urban population over the past 7 years. Public health efforts should focus on eliminating local transmission, and once eliminated, reintroduction from distant migration is unlikely.

## Author Summary

Urban transmission of schistosomiasis is becoming more recognized as rural disease is becoming less common and urbanization increases. Characteristics of infection of the immigrant population to cities and genetic characteristics of the parasite population itself indicate local transmission is the most important factor for the presence of the parasite rather than arrival of infected immigrants. While there is 70% coverage of adequate sanitation, this was insufficient to interrupt transmission. If eliminated, this focus is unlikely to readily reappear due to immigration.

## Introduction

Urbanization, the concentration of regional populations in cities, has been the great global demographic trend of the last 100 years, and the urban context has influence the nature and distribution of parasitic diseases, such as schistosomiasis. While the disease is usually thought of as a rural problem, it is increasingly recognized in large urban areas of Brazil [1,2,3,4,5,6]. In Brazil, urbanization has been rapid, and today 86% of Brazilians live in cities [7] compared with 80% of the US population [8]. The city of Salvador, the capital of the state of Bahia, is a good example of this growth. The city’s population has increased by 300% in just 20 years, mostly due to migration from rural areas. At this pace, city services have not been able to keep up in the neighborhoods with the highest proportion of informal housing [9,10]. Schistosomiasis is ultimately a disease of inadequate sanitation, so that the pace of migration may also influence the presence or persistence and even spread of schistosomiasis in the urban environment. The relative contribution of migration or local transmission, therefore, becomes an important public health issue when considering control measures.

**Fig 1:**
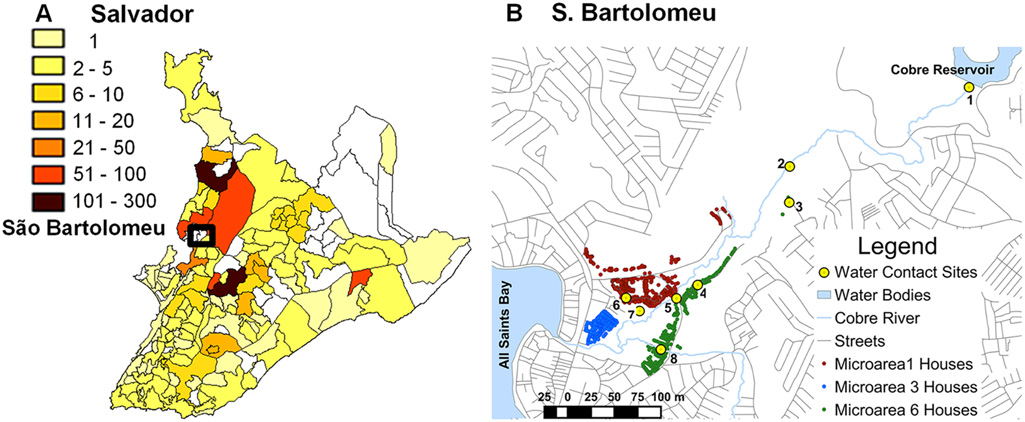
Distribution of schistosomiasis in Salvador and the neighborhood of São Bartolomeu. A. Distribution of mean annual *S*. *mansoni*-positive fecal examinations in Salvador by neighborhood 2000–2006. No information was available for blank areas. Source: Salvador Municipal Secretariate of Health (http://www.tabnet.saude.salvador.ba.gov.br). B. Map of São Bartolomeu showing homes in the 3 microareas (MA) and rivers running through the community as well as streets in surrounding neighborhoods. Water contact sites: 1. Reservoir dam; 2. Gardens; 3. Gardens entrance; 4. Fountain Street; 5. Iron Bridge; 6. São Rafael Passageway; 7. Swamp beside soccer field; 8. Manguete and Snake Streets. Sites 1, 2 and 5 were significantly associated with infection.

The presence of schistosomiasis in the city of Salvador is not a new problem. In fact, the city has had a historic role in the study of schistosomiasis. *Schistosoma mansoni* was conclusively differentiated from *S*. *haematobium* in 1908 by the Brazilian scientist, Pirajá da Silva, based on patients resident in Salvador [11]. Between 2000 and 2006, the state required reporting of all cases of schistosomiasis identified in municipal areas. All regions of the city reported cases from clinics (Fig. 1A), and multiple hospitals. Still, prevalence for the whole metropolitan area of Salvador in the last 10 years has only been between 2 to 5% [12]. Hospital cases were concentrated in the central and northern regions of the city consistent with the Municipal Health Department reports of positive stool exams and predictably included areas with the greatest combination of low income, high population density and new migration [13,14].

Some degree of local transmission has been demonstrated in the city. In 2004, 30% of school-aged children in the São Bartolomeu neighborhood of Salvador were found to have schistosomiasis [15]. Some children had stool egg counts in the thousands, where 400 eggs per gram of feces (epg) is considered heavy. Three of the 298 children examined had splenomegaly. Further, a 2011 cross-sectional survey of major water bodies in Salvador [16] found *S*. *mansoni* infected snails in 7 of 158 locations. What percentage of infections in the city is due to migration from endemic rural areas and what percentage represents local transmission is not known, but it is an important question when considering public health measures. We studied the distribution of infections within the human population and the genetic epidemiology of *S*. *mansoni* in São Bartolomeu to better understand the risks, sources and persistence of the infection within a large metropolitan area.

## Methods

### Ethics statement

The Committee on Ethics in Research of the Oswaldo Cruz Foundation of Salvador, Bahia, the Brazilian National Committee on Ethics in Research and the Institutional Review Board for Human Investigation of University Hospitals Case Medical Center, Cleveland, Ohio approved the study design. All subjects provided written informed consent or in the case of minors, consent was obtained from their guardians. All aspects of the study have been conducted according to the principles expressed in the Declaration of Helsinki.

### Study site and population

The neighborhood of São Bartolomeu (12^o^ 54′4" S, 38^o^28′31" W) is located in the northwestern part of the city between the Cobre Reservoir with its nature park and the All Saints Bay. The Cobre River drains from a large reservoir into a small mangrove swamp and then out to sea. The neighborhood surrounds this outlet of the Cobre River (Fig. 1B) and is home to nearly 5,000 inhabitants. Socially, São Bartolomeu is considered a low-income and high-crime area with housing that dates from the 1970's, but informal new housing is continually being added. A recently established unit of the Federal Family Health Program has divided the region into 6 microareas. Each has approximately 800 residents, and each has an assigned health agent who is a liaison between the community and the clinic. For comparison we used data from our study on the distribution of schistosomiasis and its genetic epidemiology in 2 rural communities 200 km southwest from Salvador [17,18] in the municipality of Ubaíra (13^o^ 14’ 59” S, 39^o^ 43’ 60” W).

### Study design and protocol

The 3 microareas with the highest prevalence of *S*. *mansoni* infection in school-aged children in 2004 [15] were selected for study. All households were visited, numbered and their location registered with a hand-held Trimble. Nomad GPS unit (Model 65220–11). Responses to a questionnaire were recorded for each resident. Interviewers solicited the respondent’s age, sex, race, years of residence in Salvador, previous residence, travel outside of the city of Salvador, education, occupation, major household goods, use of city services, frequency of flooding, history of *S*. *mansoni* infection, treatment for schistosomiasis, surface water contact sites commonly visited and activities performed at these sites. Parents and guardians responded to some questions for minors <12 years of age. Water contact information was obtained directly from each resident. The 8 major sites where residents came into contact with surface water were identified by community members and confirmed in the survey. All contact sites were located along the Cobre River (Fig. 1B).

### Field laboratory procedures

All residents >4 years of age were asked to provide 3 stools on different days for parasitologic examination. We also analyzed stool eggs from 9 infected residents of São Bartolomeu collected in 2004 for comparison. For fecal examination, the stools were weighed to the nearest 0.1 g with a digital balance (Startools, China), and then a single slide was processed by the Kato-Katz method and examined microscopically to identify ova of *S*. *mansoni* and other helminths. The number of *S*. *mansoni* ova were quantified, recorded and finally expressed as eggs per gram of feces (epg). Total egg counts were calculated as stool weight X epg. Each stool positive for *S*. *mansoni* ova was homogenized, filtered and sedimented as described [18] to obtain a sample enriched for these ova. A standard phenol-chloroform extraction was followed by treatment with hexadecyltrimethylammonium bromide (CTAB) to remove PCR inhibitors [19].

### Microsatellite genotyping

Primers for 15 microsatellite markers [20] were used to amplify *S*. *mansoni* DNA. The specificity of these markers has been demonstrated for this parasite in stool samples as have the utility [18,20,21,22] and limitations [23] of the approach for schistosomes as well as other parasites [24,25,26]. The intensity of resultant amplicons was measured by automated sequencer and analyzed with the program Peak Scanner (Applied Biosystems, Waltham, Massachusetts). Allele frequencies were calculated by dividing the peak height for each allele by the sum of all peaks for the microsatellite. Peaks providing less than 5% of the total intensity were excluded as were markers where no peaks were greater than 100 pixels using the program’s default settings. The number of alleles for each marker was calculated by multiplying the allele frequency by sample total egg counts.

### Data analysis

Demographic data and water contact were analyzed for their association with infection status using logistic regression or for infection intensity with forward conditional linear regression in SPSS. Independent variables were age, sex, migration status, percent of lifetime spent in the community, income, household size, type of sanitation, history of household flooding, number and location of water contact sites visited, previous *S*. *mansoni* infection and history of treatment. Age groups at 5-year intervals were compared for prevalence of infection. Adequate sewage disposal was defined as the presence of a connection to the municipal sewer system or a septic tank. Household density was evaluated as number of occupants per number of bedrooms. Persons born outside of the city of Salvador were considered immigrants. p values <0.05 were considered statistically significant if less than the value obtained after Bonferroni correction for multiple comparisons. **Infrapopulations** were defined as all of the parasites within one host. **Component populations** were all the parasites within a host species usually within a limited geographic area. We also stratified parasites as component populations based on host demographic characteristics.

**Fig 2:**
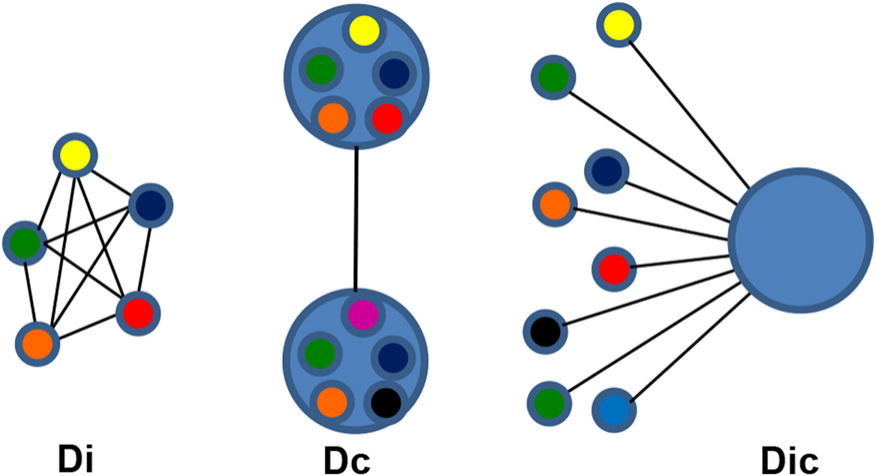
Schematic diagram of population comparisons for differentiation. **Di**—Small circles represent infrapopulations infecting a single host. The individuals in the infrapopulations are heterogeneous, but they are all represented by a single color, their average allele frequency. Each line is a discrete value for the pairwise Jost’s D between infrapopulations. The Di’s of infrapopulations from a group of hosts are summarized by the average. **Dc**—Large circles represent component populations made up of all the parasites in an area or a group of hosts with the same epidemiologic characteristic. The Dc is calculated from the combined allele numbers for all members of the component population compared to the allele numbers from the comparison group. From a comparison of the 2 groups, a single value for Jost’s D is calculated. **Dic**—This compares the allele frequencies of individual infrapopulations to the combined allele frequency of their source component populations. Each infrapopulation will be characterized by a single value for Jost’s D that represents its relative similarity to the component population. The Dic’s of infrapopulations from a group of hosts are summarized by their average.

Genetic differentiation based on Jost's D [27] was used to compare pairwise the infrapopulations of infected individuals (Di). The mean for all Di comparisons was calculated for stratified subsets of infected individuals based on demographic, geographic or parasitologic characteristics (Fig. 2). When the combined allele numbers for infrapopulations from one group of hosts were compared to a different group, this comprised the component population differentiation (Dc). Each individual's parasite infrapopulation was compared to the component population to yield the Dic. The Dic produces a single number representing how differentiated an individual's population is from the population infecting all humans in São Bartolomeu. Jost’s D and its confidence interval were calculated with the Diversity Index function in the Species Prediction and Diversity Estimation program (SPADE) (http://chao.stat.nthu.edu.tw, Chao, A. and Shen, T.-J., last accessed 6–9–2013). Finally, the effective allele number (AE), a measure of genetic diversity, was calculated as described [16,28]. Means for continuous variables were compared by a bootstrapped Student's t-test using SPSS for Windows (Version 19.0). Egg counts were normalized by log-transformation. Supplemental data (S1 Dataset) containing demographic data and infrapopulation allele frequencies have been deposited with Case Western Reserve's online source for curated digital content, Digital Case (**http://library.case.edu/digitalcase/DatastreamListing.aspx?PID=ksl:blanton2**).

## Results

### Study population

A total of 1,335 residents were identified in the 3 selected microareas (MA1, MA3, and MA6) of São Bartolomeu, and 91% were enrolled in the study. The mean per capita income for the study population was R$325 ($140) compared to a mean of R$1496 ($643) for the city of Salvador [29]. Municipal water was supplied to 99.9% of the homes and 67% used either a septic tank or the municipal sewer system (Table 1). Although the whole area of São Bartolomeu is less than 1 km^2^, the sample characteristics of MA3 were significantly different than the other MAs. A higher proportion of residents of MA3 were born outside of Salvador, had lower coverage with adequate sanitation, and flooding was more common (Table 1). Of the current residents, 22% were born outside of the metropolitan area of Salvador. The majority of immigrants were from within the state (92%) and came from a median distance of 178 km from Salvador. Immigrants were significantly older (44.1 ± 16.7) than natives (24.6 ± 15.4).

**Table 1:** General characteristics of the studied population in São Bartolomeu.

		Total	MA1	MA3	MA6	pMA_1,3,6_ ^a^
**Characteristic**		n = 1228	n = 439	n = 335	n = 447	
**Infection**	Prevalence (%)	300 (24.7)	101 (23.1)	81 (24.3)	118 (26.5)	0.51
	Intensity^c^ (SD)	60.8 (4.6)	50.3 (3.0)	69.7 (5.0)	64.9 (4.9)	0.27
**Male Sex (%)**		554 (45.1)	187 (42.6)	157 (46.9)	206 (46.1)	0.43
**Mean Age (SD)**		29.2±17.8	28.6±18.0	29.0±17.5	29.8±17.7	0.43
**Birth place (%)**	Salvador	946 (77.6)	355 (81.2)	232 (69.3)	359 (80.3)	**<0.001**
**% Life in Salvador (SD)**		90.7 (20.2)	92.1 (18.9)	87.7 (22.0)	91.6 (19.7)	0.01
**Piped water (%)**		1080 (92.5)	394 (94.7)	309 (92.8)	377 (90.2)	0.89
**Sanitation (%)**	Indoor Toilet	1029 (95.9)	386 (98.7)	294 (96.4)	349 (92.6)	**<0.001**
	Septic tank/ Sewer	711 (67.2)	303 (78.9)	167 (56.2)	241 (63.9)	**<0.001**
	River/Open air	347 (32.8)	81 (21.1)	130 (43.8)	136 (36.1)	
**Flooding**		601 (49.4)	170 (39.0)	224 (67.1)	207 (46.4)	**<0.001**

^a^pMA—the p-value by chi-squared analysis for the comparison of the subscripted microareas.

^b^Significance for 2X3 table.

^c^Geometric mean egg counts per gram of feces

Bold type indicates significant p values after Bonferroni correction for multiple tests.

### Infection and risk factors

**Fig 3:**
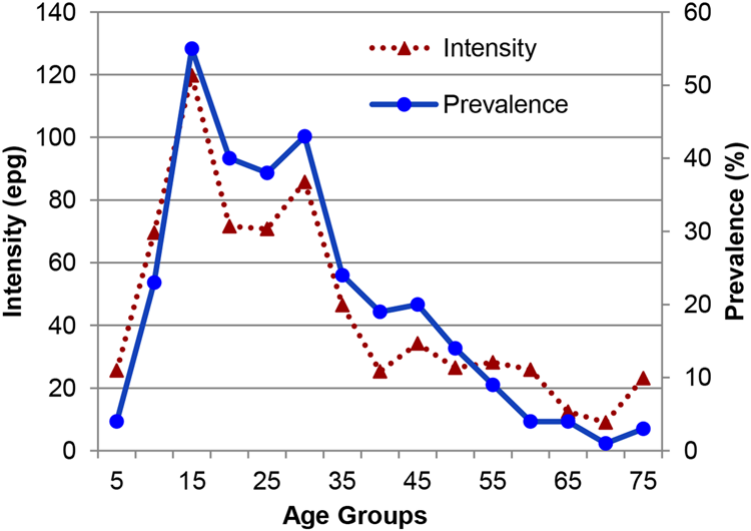
Geometric mean intensity and prevalence of *S*. *mansoni* infection in 5 year intervals. Mean intensity was calculated only for those who were egg positive.

Out of a population of 1,335, 92% (1,225) participated in the parasitologic survey. Of these 82% provided 3 stools, 10% furnished 2 stools and 8% gave 1 stool. In total, 300 (24.7%, Table 1) had *S*. *mansoni* ova on examination. Univariable analyses showed that male sex, age group, prior infection or treatment for schistosomiasis and number of water sites visited were risk factors for current schistosome infection. Trips outside of Salvador, an adequate sewer system, low household density and reported lack of contact with surface water collections were negatively associated. The prevalence and intensity of infection with *S*. *mansoni* was highest in the 11–15 age group, and when compared to the 5-year-old age group, prevalence remained significantly higher in older groups up to the age of 45 (Fig. 3). In multivariable analysis, sex, household density and number of water contact sites visited remained independently associated with infection, while increasing number of visits outside Salvador and immigrant status were not (Table 2).

**Table 2:** Risk factors for *S*. *mansoni* egg positive stools.

**Variable**		**Total**	**n infected (%)**	**p value**	**OR (95% CI)**
**Sex**	Male	554	199 (66.3)	**<0.001**	2.47 (1.80–3.38)
	Female	671	101 (33.7)	-	-
**Persons/Bedroom>2**	Yes	540	89 (23.5)	**0.003**	1.61 (1.17–2.21)
	No	488	154 (40.0)	-	-
**Water Contacts**	0–9	1225	300 (26)	**<0.001**	1.28 (1.20–1.37)
**Age (range, median)**	4–94	27	300	0.327	1.00 (0.99–1.01)
**Outside Trips Last Year**	0–3	1225	76 (19.0)	0.012	0.71 (0.54–1.91)
**Immigrant**	Yes	274	50 (18)	0.389	1.22 (0.78–0.78)
	No	951	250 (26)	-	-

Variables were first tested for association or correlation with infection by univariate analysis and then significant variables were used in logistic regression analysis to identify those that were independently associated. Significant p values following Bonferroni correction are in bold.

Immigrants were considered those not born in Salvador. They were substantially older than natives (17 years on average), and had lower prevalence (18.2% vs 26.5%, p<0.01) and intensity of infection (40.2 vs 65.7 epg) than those born in Salvador. Controlling for age and sex, the percent of lifetime immigrants spent in Salvador was significantly associated with infection, although the effect size was small (p = 0.011, OR 1.019, CI 95% 1.004–1.034).

**Fig 4:**
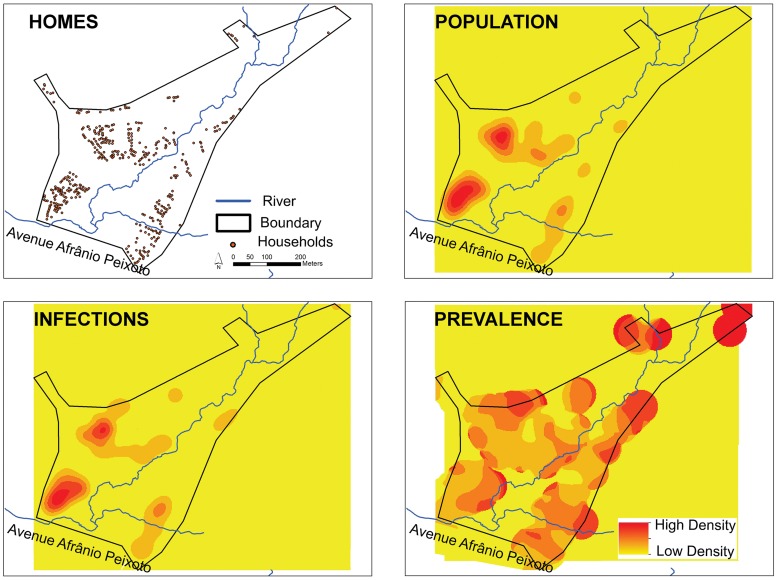
Kernel density analysis of the prevalence of *S*. *mansoni* infection in São Bartolomeu. The spatial unit of reference is the home and each participant in the study was geo-referenced with their respective positive or negative parasitological result. Density analyses were generated with use of the Kernel density estimator implemented in the software ArcGIS spatial analyst extension 10.1. Density analyses were generated with bandwidth (search radius) of 50 meters. The location of **Homes**, the location and number of the human **Population**, the location and number of **Infections** and the **Prevalence** density (Infections/Population) are shown.

Three water contact sites were significantly associated with infection prevalence (Table 3). The OR’s were 1.91–2.15 for contact with these sites. Contact site 1 is at the outlet for the Cobre Reservoir Dam. The site is relatively shallow and is used for netting fish. Site 2 was used for crossing small streams within an area used for small-scale commercial vegetable production along the flood plain of the river. Contact site 5 is located at a bridge that spans the river on a road that joins MA’s 1 and 6. The bridge is a gathering point for young people as well as used for fishing. Point 7 is a vegetation-covered wetland near a soccer field. A kernel density map suggests that risk of infection increases with distance from the major avenue that borders the neighborhood (Fig. 4), although the population density and absolute number of those infected is higher near this thoroughfare.

**Table 3:** Risk at contact points for *S*. *mansoni* infection.

**Contact point**	**Contact(%)**	**Infected(%)**	**OR (95% CI**	**p-value**
**1**	210 (17.3)	103 (48.4)	**2.14 (1.48–3.09)**	<0.001
**2**	245 (20.1)	112 (45.5)	**2.10 (1.33–3.30)**	0.001
**3**	270 (22.2)	107 (39.5)	0.71 (0.44–1.16	0.168
**4**	308 (25.3)	123 (39.8)	1.16 (0.77–1.74)	0.484
**5**	354 (29.1)	153 (43.1)	**2.28 (1.62–3.22)**	<0.001
**6**	297 (24.4)	96 (32.1)	0.73 (0.50–1.07)	0.103
**7**	370 (30.4)	130 (34.8)	**1.65 (1.18–2.31)**	0.004
**8**	237 (19.5)	93 (39.1)	1.14 (0.80–1.64)	0.460
**Other**	571 (47.0)	165 (28.6)	1.02 (0.77–1.37)	0.875

1. Reservoir dam; 2. Gardens; 3. Gardens entrance; 4. Fountain Street; 5. Iron Bridge; 6. São Rafael Passageway; 7. Swamp beside soccer field; 8. Manguete and Snake Streets

### Parasite population differentiation

The average differentiation between replicate samples is 0.007. There was little genetic differentiation (Dc) between the parasite populations when stratified into component populations based on host sex, age, infection intensity or geographic location within São Bartolomeu (Table 4). Geographically, the Dc between the parasite population in São Bartolomeu and the rural area of Ubaíra Dc was 0.103. A temporal comparison was made between samples collected in a 2004 pilot study from 9 infected children in São Bartolomeu and the current component population. The Dc for these samples was 0.007.

**Table 4:** *S*. *mansoni* subpopulation differentiation and diversity.

		**Dc**	**Di (p-value)**	**Dic (p-value)**	**Ae (p-value)**
**All**	Host sex (male vs female)	0.003	0.053 vs 0.071 (**<0.001**)	0.029 vs 0.039 (0.058)	3.360 vs 3.305 (0.051)
	Host age (≤15 vs >15 y/o)	0.003	0.045 vs 0.063 **(<0.001**)	0.024 vs 0.034 (0.018)	3.329 vs 3.351 (0.341)
	Intensity of infection (<400 vs >400 epg*)	0.001	0.065 vs 0.023 (**<0.001**)	0.036 vs 0.012 (**<0.001**)	3.308 vs 3.506 (**<0.001**)
	Contact point 1 (Yes vs no)	0.002	0.051 vs 0.062 (**<0.001**)	0.028 vs 0.034 (0.230)	3.341 vs 3.347 (0.255)
	Contact point 2 (Yes vs no)	0.002	0.043 vs 0.068 (**<0.001**)	0.023 vs 0.038 (**0.002**)	3.339 vs 3.348 (0.197)
	Contact point 5 (Yes vs no)	0.003	0.047 vs 0.070 (**<0.001**)	0.026 vs 0.039 (0.008)	3.362 vs 3.325 (0.038)
	MA1 vs MA3	0.005	0.069 vs 0.056 (**<0.001**)	0.038 vs 0.030 (0.170)	3.284 vs 3.400 (0.042)
	MA1 vs MA6	0.001	0.069 vs 0.042 (**<0.001**)	0.038 vs 0.023 (**0.004**)	3.284 vs 3.390 (0.048)
	MA3 vs MA6	0.006	0.056 vs 0.042 (**<0.001**)	0.030 vs 0.023 (0.140)	3.400 vs 3.390 (0.791)

Di—pairwise Jost's D for all members of the group. Dic—mean Jost's D for each infrapopulation in the group compared to the village component population. Ae—mean effective allele number. Bootstrapped Student's t-test was used to compare group means for these indices. Dc—Jost's D for the component population formed by the total allele numbers for the group. Comparisons significant after Bonferroni correction are indicated in bold. Other variables tested but without significant differences were trips outside the region, co-infection with other helminths, all other water contact points, number of water contacts visited, a history of past infections. MA1, MA3, MA6—microarea divisions of São Bartolomeu. See Fig. 1. * epg—mean count of *S*. *mansoni* eggs per gram of stool.

Within São Bartolomeu, the Di different for all comparisons. The large number of eggs makes p values less useful and confidence intervals will not overlap. The largest difference, however, is for intensity of infection greater or less than 400 epg (Table 4). The Dic was statistically smaller for those more heavily infected, those visiting site 2 and for those living in MA6 compared to MA1. While there was no statistically significant difference for the mean prevalence and intensity between these microareas, MA6 had 5 of the 10 most heavily infected individuals, whereas, MA1 had only 1 of the top 10 intensities. The effective allele number (Ae) was significantly higher for those more heavily infected.

### Immigrant and native parasite population structure

The Dc treats all the parasites in 2 groups of hosts as a single population. The DC in urban São Bartolomeu was very low between natives and immigrants suggesting infection from a common source. In the rural area, the wider DC between natives and immigrants may indicate some contribution from different sources. The rural area, however, does include 2 sites separated by 8 km. The Di and Dic, which represent comparisons based on individual infrapopulations, were significantly different between parasite populations of native born and immigrant residents of São Bartolomeu (Table 5), but not between those of natives compared to immigrants of the rural area. A positive correlation between the average pairwise Di and the log epg was weak but significant (r^2^ = 0.08, p<0.01) and stronger between the Dic and log epg (r^2^ = 0.23, p<0.01). The Ae was lower in the urban site compared to the rural area, but did not differ between natives and immigrants for either area.

**Table 5:** Parasite population differentiation in immigrant and native populations.

		**Dc**	**95% CI**	**Di**	**p value**	**Dic**	**p value**	**Ae**	**p value**
**São Bartolomeu**	Native	-	-	0.058	<0.001	0.031	0.038	3.016	0.99
	Immigrants	-	-	0.108	-	0.059	-	3.012	-
	Native vs Immigrants	0.002	0.001–0.004	-	-	-	-	-	-
**Ubaíra**	Native	-	-	0.124	0.082	0.068	0.416	3.351	0.96
	Immigrants	-	-	0.128	-	0.074	-	3.349	-
	Native vs Immigrants	0.022	0.017–0.026	-	-	-	-	-	-

Dc—Jost's D for the component population composed of all members of the group. Di—mean pairwise Jost's D between infrapopulations for all members of the group. Dic—mean Jost's D for each infrapopulation in the group compared to the community component population. AE—mean effective allele number. CI 95% determined with 1000 bootstrap replications using the program SPADE. Student's t-test was used to compare group means for these indices.

## Discussion

Multiple Brazilian cities have seen outbreaks of schistosomiasis [4,6,30,31,32,33]. In some cities like Salvador, this is not so much a new introduction as it is a low level continuation of a pattern of infection present for some time [34,35,36]. Although Bahia is a state where schistosomiasis is endemic, Salvador, its capital, is considered a non-endemic area. This would be a valid designation if the infection were not transmitted and only found in immigrants to the city, but this is not the case. The overall prevalence is only 2–3% [36], but it can exist in islands of transmission where the community-wide prevalence is >20% and even cases of hepatosplenomegaly can be identified [15]. Given the rapid growth of the city, it is important to understand the relative contributions of immigration and local transmission to the presence of the infection.

Several epidemiologic indicators suggest local transmission is a more significant driver for *S*. *mansoni* infection in the neighborhood of São Bartolomeu than immigration. Guimarães et al. [15] found in 2004 that most infected children in São Bartolomeu had never left the area even for visits or vacations and also identified infected snails. In this study we extended this analysis to include all inhabitants in selected sections (microareas) of the neighborhood. Immigrants were less likely to be infected than natives (Table 2), and infection in immigrants increased with increasing time spent in São Bartolomeu.

Parasite population genetic structure and history also suggest local transmission was the major source of parasites. We and others have previously observed a great diversity in rural populations of schistosomes and a degree of reproductive isolation over even short distances [18,37]. Immigrants, particularly newer immigrants, infected at distant sites would be expected to carry strains that are genetically differentiated from those transmitted at their current residence. For São Bartolomeu, the overall mean pairwise Di was 0.063, which was lower than that we observed in the rural area of Ubaíra where the mean Di's were 0.097 [18]. Comparing the Di for those born in Salvador with those who immigrated there (Table 5), there is a nearly 2-fold difference, but this is likely due to the immigrant’s lighter infection and not to the introduction of a heterogeneous population. Factors associated with an increase in the relative sample size of parasite eggs tend to be associated with a relative decrease in Di and Dic. The younger age, higher prevalence and intensity for the native born in the urban area are consistent with having lower Di and Dic than immigrants. By contrast, immigrants to the rural area did not differ for these characteristics or for these differentiation indices. The Dc, however, indicates that both natives and immigrants were infected from the same pool of parasites, especially in the urban area. The Dc in the rural area was higher than the mean differentiation between replicate samples (0.007) and may indicate a higher degree of introduction of parasites from a distant population or populations.

Finally, comparing the parasite population in 2004 to that in 2011, the Dc is near the replicate error rate. Its genetic composition has changed little in 7 years. The lack of Dc differentiation by age also suggests that the parasite population is stable and little influenced by migration. The combined genetic characteristics of *S*. *mansoni* in São Bartolomeu indicate there is a single parasite population with no internal obstacles to gene flow and few new introductions of genetically diverse parasites. It is likely that most of today’s children as well as adults became infected in São Bartolomeu, and the prevalence there is not being sustained by the arrival of people infected far from Salvador. A drawback for using pooled samples is that null alleles cannot be accounted for. This should have little effect on differentiation, since the risk of these should be equally distributed between the comparison groups.

The age distribution, prevalence of infection, association with a perennial water source and poor sanitation make transmission in the urban site similar to many rural sites in Brazil. Having more than 3 people per bedroom as a risk factor may reflect socio-economic development, although crude household income itself was not associated with infection status. The number of water contact sites regularly visited was used as a proxy for the amount of water contact. This relies on recall and could be subject to recall bias. The kind of recall is relatively coarse and was not influenced by interviewers knowing infection status. In the urban and the previously studied rural area [17], increasing numbers of sites visited was associated with increasing risk for being infected.

One of the factors that makes infections like schistosomiasis unexpected in cities is the presence of city services and sanitation. Worldwide, drinking water is a first priority in municipal development before sanitation, and essentially everyone in São Bartolomeu has municipal water piped to his or her home. For Salvador, at the turn of the 20th century, a series of reservoirs were constructed outside what was then the city limits. As the city expanded rapidly after World War II, these became surrounded by new housing with poor sanitation such that many of these collections became polluted and the waterways used to occupy new areas became open sewers. São Bartolomeu is downstream from one of these early development projects. The Cobre Reservoir is still relatively protected, while it’s outflow, the Cobre River, is not. It is a recipient of raw sewage not only from the São Bartolomeu community itself but all from the densely populated hills surrounding it. The city of Salvador has grown so rapidly that attempts to keep up with the infrastructure requirements around it can be described as heroic. In 2004, a citywide program for the introduction of sewer systems increased the coverage from 40% to 70% in São Bartolomeu. The Bahia Azul Project, as it was known, was demonstrated to have an enormous effect on reducing the incidence of diarrheal diseases in the city [38]. The effect on schistosomiasis in this area, however, appears to have been negligible. The prevalence of infection is the same in children today as in 2004 despite a degree of coverage by the municipal sewer system superior to many emerging countries of the world. Nevertheless the 70% coverage is not sufficient where raw sewage makes its way to waterways that large numbers of people use for recreation and commercial activities. The persistence of schistosomiasis represents a failure of city services. Fortunately, our analysis indicates transmission in the city is focal and elimination of these islands of infection should produce long-term control despite migration [16].

## Supporting Information

S1 Dataset Excel file is provided tabs for variable key, deidentified demographic data used in this paper and infrapopulation allele numbers. (XLSX)

S1 Checklist STROBE checklist. (DOCX)
